# The Organon and Materia Medica Club of the Bay Cities of California

**Published:** 1894-12

**Authors:** W. E. Ledyard

**Affiliations:** Secretary


### THE ORGANON AND MATERIA MEDICA CLUB OF THE BAY CITIES OF CALIFORNIA.

### Meeting of September 7th, 1894.

Sections 21 and 22 of *The Organon* were read and discussed.

Dr. J. M. Selfridge—How did Hahnemann arrive at the conclusion that "the morbid symptoms which medicines produce in healthy persons are the sole indications of their curative virtues in disease"?

Dr. McNeil—Hahnemann who had been cured of "chills and fever" by Cinchona Bark, noticed that the *latter was capable of producing similar symptoms in the body in a state of health.* The effects of *vesicular erysipelas* and of *poison oak* can be distinguished from each other, only by the history of the case.

Dr. Wilson—I had to give up farming, on account of the effects |produced by the *poison ivy:* A watery exudation, with symptoms resembling erysipelas, œdematous swellings, etc.

Dr. McNeil—Hebra, while lecturing on so-called skin diseases, applied *Croton Oil* to the surface, to produce an eruption to illustrate the subject.

Dr. Swayze—Reported the case of a woman who was *vaccinated* by cow-pox virus. An *ulcer* on the toe followed. This was treated by a *salve,* and healed up, but *another formed higher up.* The above treatment was repeated with the same result, the ulcer *forming higher* and *higher up each time.*

Secretary—This is just the reverse of the *curative process* which proceeds *from above downward.*

Dr. Swayze—The ulcer was very *deep;* she had the "*pinched off*" stool of *Causticum,* which relieved, while there was a *decided aggravation from Silicea,* a remedy that frequently removes the bad effects of vaccination. These two remedies were followed by beneficial results.

Dr. J. M. Selfridge—To show the susceptibility of some people for drugs, reported the case of a woman *who could not uncork a Rhus-tox.*$^{3x}$ *vial, without being poisoned.*

Dr. McNeil—Reported the case of a patient who can at once detect a very high potency of *Natrum-mur.* She is subject to *Natrum-mur. headaches* and needs that remedy.

Dr. Swayze—Will cases so susceptible to drug action be cured by the remedy to which they are so susceptible?

Mr. Tapley—Proved his own susceptibility to *Psorinum,* a dose of which produced *a purulent discharge from the eye; a rainbow before the field of vision,* and other symptoms, some of which continued for three months.

Dr. J. M. Selfridge—Referred to a patient in whom *Bryonia* always produced pathogenetic symptoms.

The Secretary knew of several patients in whom *Sulphur* and *Nux-vomica high* had a similar effect.

Dr. J. M. Selfridge—My preceptor was very susceptible to the action of *Ipecac.* He always knew when I had been mixing *Dover's Powder,* which contains *Ipecac.*

Dr. McNeil—The allopaths recognize idiosyncrasies as they relate to drug action. We can see the *paralytic tendency, especially in the coal-tar derivatives,* which they are gradually discarding on that account. In alluding to the bad effects following *the grippe,* when treated allopathically, he asserted that it *was not the grippe that killed, but the treatment.*

Mr. Tapley—Remarked that Dr. Swan stated *that eighty-two deaths occurred in one day in New York,* when *the grippe was treated by Quinine.*

Dr. Augur—When an allopath, *treated grippe with fifteen-grain dose of Antipyrin,* which at once relieved the headache and backache. However, *he now believes that such treatment lessens the resisting power of the vital forces.*

Dr. McNeil—Stated that when he had the *grippe,* there was a *terrible aching all over.* After taking the indicated remedy, which he has found to be either *Rhus or Bryonia,* he was ready for any duty in twenty-four hours.

Dr. Swayze—Has found *Cadron* to relieve in cases of *the grippe.*

Mr. Tapley—In cases attended with great soreness, cured with *Arnica.*

Dr. Swayze—In rheumatism, with extremely *sour sweat,* and

*a sensation as if he were lying on a bed of rocks* (*similar to Arnica*), cured by *Pyrogen*$^{cm}$.

Then followed a discussion on "*Nosodes.*"

Dr. McNeil—Thinks they should all be prepared from the original.

Dr. Swayze—Referred to cases in which *Medorrhinum* relieved *light*-complexioned persons and failed to relieve those who were *dark*.

Mr. Tapley—Cured an *ulcer* by giving *the morbid product, followed by Pyrogen.* He reported a case of *suppressed eruption* and *symptoms of consumption:* emaciation, cough, profuse expectoration, night-sweats, etc., cured by *Sulphur*, *Calcarea*, and *Lycopodium*, in the CM *potency*. The eruption *reappeared and the symptoms of consumption disappeared.*

Dr. McNeil—Has cured several cases *antotoxically.*

Secretary—Has successfully treated the effects of a *bee-sting* with a dose of *Apis*$^{200}$; poisoning by strong *Ammonia-carb.*, with a dose of *Ammonia-carb.*$^{cm}$ (Swan), and *Rhus* poisoning by *Rhus-tox.* in potencies ranging from the 200th to the DMM.

Dr. Swayze—Had a case in which the prepuce was much swollen, with a sensation in the urethra of something flapping back and forth, with terrible *itching* and *soreness* and *cutting after micturating;* cured by *Argentum-nitricum.*

Dr. McNeil—Asserted that a *gleety* case must be treated by the indicated antipsoric. *He treats recent cases of gonorrhœa with the epidemic remedy. Rhus-tox.* or *Bryonia* has been the *epidemic remedy* for the last ten years. *It is the remedy for all acute diseases arising from atmospheric influences.*

Secretary—Suggested that *Rhus-tox.* was indigenous to California, and that that might account for its being the epidemic remedy in this country.

Dr. J. M. Selfridge—Has had cases of *pneumonia* in which no *remedy but Phosphorus would cure.*

Dr. McNeil—The allopaths have discovered that *scrofula* and *tubercle* are identical.

(Signed) W. E. LEDYARD,
*Secretary.*

### SEPTEMBER 21ST, 1894.

Sections of *The Organon* 23 to 28 and 58 to 62 and 69 were read and discussed.

Dr. McNeil—Speaking of the treatment of *burns*, reported a case in which the patient was burnt very extensively while sleeping near a fire. After being put into *hot* water there was absolute freedom from pain. She had previously complained much of being chilly. Hebra tells of keeping a patient suffering from the effects of a burn in a *hot* bath for one hundred and seventy days.

Dr. C. M. Selfridge—Suggested a solution of *Cantharides.*

Dr. J. M. Selfridge—Advocates an *Isinglass plaster* as the very best local application in case of a burn.

Dr. McNeil—Would use the *hot* bath and *the indicated remedy.*

Dr. Augur—Is in favor of *Carbonate of Soda* dusted on the burnt part.

Dr. J. M. Selfridge—In large burns advises *sugar* (yellow C) to be sprinkled over the parts.

Dr. Swayze—Has recourse to lamb's wool in similar cases.

Dr. McNeil—Remarked that the "Old School" physician had been stealing our thunder. He would pick out a cathartic or an emetic which would produce *not the same kind of diarrhœa or vomiting*, but *any carthartic or emetic.* As a matter of course he has abandoned this false Homœopathy.

Dr. Swayze—Desired to have the opinion of the Club with regard to the use of *Rhus-tox.* in *treating Poison Oak.*

The Secretary—Has frequently witnessed the beneficial effects of such treatment.

Dr. McNeil—Stated that *Graph., Mez.*, and other remedies had been recommended. He worked out a case in his own person by means of *Bœninghausen's Repertory* and relieved himself in a short time with *Sulphur*, which was the indicated remedy in his case. He turned to the chapter on "Relationships of Remedies," in the above repertory, to the Section on "*Rhus*" and to the sub-section "*Skin*," in which is a list of remedies capable of producing *similar* eruptions. From the list he would select *the most similar remedy*, exactly as in the treatment of any other case *without regard to the name of the disease.*

Dr. J. M. Selfridge—Has relieved the symptoms of *Rhus poisoning* in his own person by *Yerba Santa.*

Dr. Swayze—Made the statement that *Grindelia Robusta* is sometimes indicated.

Mr. Tapley—Reported a case of *Poison Oak* with symptoms resembling *rheumatism.* He had been poisoned years before and was frequently laid up on this account. After the administration of *Rhus* he had no "rheumatism" for months.

The Secretary—Suggested that cases of so-called "rheumatism" were frequently nothing but cases of *Rhus poisoning* in which the eruption had been suppressed, either by neglect of treatment or by maltreatment. He has frequently given *Rhus* to persons susceptible to the effects of the poison and exposed to it, and given thus it seemed to act as a preventive.

Dr. Wilson—Had the same experience.

Dr. McNeil—Stated that the first great duty of the physician is to find the symptoms, and the next to find the indicated remedy.

Dr. Augur—Asked if in a chronic case *a single dose* of the indicated remedy should be given and the patient told to return in a week or two.

Dr. McNeil—Replied that he was leaning that way. He stated that a repetition of the remedy breaks up the continuity of the drug's action. He reported a case with *chilliness, aching all over, irresistible desire to move,* etc., in which he gave *one dose of Rhus-tox.*, and discharged this case of *incipient malarial fever* cured in three days. He also referred to a case of *blindness,* for which, without examining particularly into the case, *a dose of Sulphur* was given. *This partially restored the sight,* but, *after being repeated,* the *blindness returned.* What is called a relapse is often *merely the exhaustion of the remedy.* He sometimes *gives three doses an hour apart,* stopping as soon as he gets an action.

W. E. LEDYARD, *Secretary.*

